# Transformation of the Food Sector: Security and Resilience during the COVID-19 Pandemic

**DOI:** 10.3390/foods10030497

**Published:** 2021-02-25

**Authors:** Cennet Pelin Boyacι-Gündüz, Salam A. Ibrahim, Ooi Chien Wei, Charis M. Galanakis

**Affiliations:** 1 Food Engineering Department, Faculty of Engineering, Adana Alparslan Turkes Science and Technology University, 07059 Adana, Turkey; cennetpelinboyaci@gmail.com; 2 Food and Nutritional Sciences Program, North Carolina A&T State University, Greensboro, NC 27411, USA; ibrah001@ncat.edu; 3 Chemical Engineering Discipline, School of Engineering, Monash University Malaysia, Bandar Sunway 47500, Malaysia; ooi.chien.wei@monash.edu; 4 Monash-Industry Palm Oil Education and Research Platform (MIPO), Monash University Malaysia, Bandar Sunway 47500, Malaysia; 5 Research & Innovation Department, Galanakis Laboratories, 73131 Chania, Greece; 6 Food Waste Recovery Group, ISEKI Food Association, 1190 Vienna, Austria

**Keywords:** food systems, panic buying, food shortage, food waste, food loss, sustainability, food supply chain

## Abstract

The ongoing COVID-19 pandemic has resulted in a new era in the efficacy of the food supply chain, while the consequences of this new era on humanity, the economy, and the food sector are still under examination. For example, food security is one vital aspect of food systems which is directly affected. This review summarizes food security during epidemics and pandemics before moving on to panic buying, food shortages, and price spikes observed during the current crisis. The importance of food resilience, together with the need for addressing issues related to food loss and food waste, is underlined in the review towards food security and sustainable development. As a result, the pandemic has shown that our food systems are fragile. Since the global population and urbanization will grow in the coming decades, pandemics will likely occur more often, and climate change will intensify. Consequently, there is a need to ensure that our food systems become more sustainable and resilient. To that end, we have highlighted the need to develop contingency plans and mitigation strategies that would allow a more rapid response to extreme events (e.g., disasters from climate change) and transform the food sector by making it more resilient.

## 1. Introduction

A sufficient amount of nutritious and safe food is necessary for sustaining life and promoting good health. However, as the world population increases, more efforts and innovations are needed in order to feed the population. Therefore, it is necessary to increase agricultural production sustainably, improve the global supply chain, decrease food waste and loss, and ensure that all people have access to nutritious food [1]. According to the Food and Agriculture Organization (FAO) of the United Nations, “*Food security exists when all people, at all times, have physical and economic access to sufficient, safe and nutritious food that meets their dietary needs and food preferences for an active and healthy life*.” This definition points to the different dimensions of food security, including food availability, access, utilization, and stability of food supplies at global, national, and local levels [2]. The concept of stability refers to both the access and availability dimensions of food security, and within this context, the population must have access to enough food at all times. Access to adequate food must be reliable, and therefore, people should not risk losing access to food due to sudden unexpected climate, health, or economic crises. Currently, the world is struggling to fight a health crisis: The COVID-19 pandemic.

**Figure 1:**
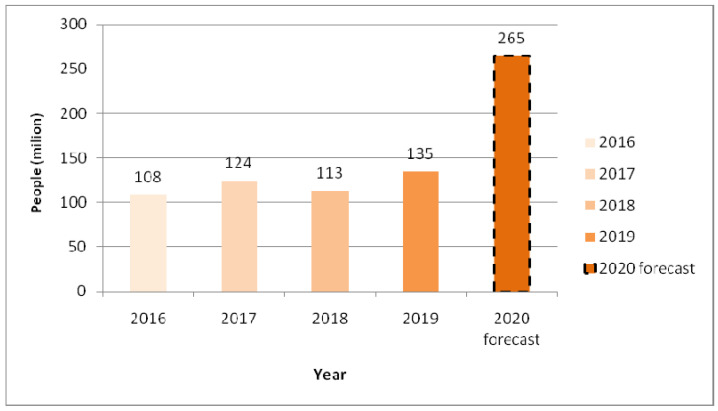
The number of people facing acute food insecurity. Adapted from [5].

The pandemic represented a sudden psychological, economic, and partly physical disruption to markets, societal sub-systems, and citizens. Food security is among the four pillars of the food systems affected in the pandemic era [3], while the latest is additional exacerbating an ongoing nutrition crisis [4]. In 2019, almost 135 million people faced critical levels of acute food insecurity or worse. The number of people in 2019 was the highest in the 4-year existence of the Global Report on Food Crises [5], as shown in Figure 1. According to the United Nations World Food Program, the number people who deal with food insecurity could nearly double to 265 million at the end of 2020 due to the economic fallout of COVID-19 [6,7]. Unfortunately, the pandemic poses a potential threat to the Sustainable Development Goals and especially, the two food-security dependent goals, no poverty and zero hunger, will be hit hard during the lockdown period, particularly in developing countries [7].

The COVID-19 crisis has already changed food systems through its effects on demand, food supply, and capacity to produce and distribute food, the behavior of consumers such as panic buying, shortages in some food groups, and food waste and loss. Therefore, COVID-19 impacts all four elements (availability, stability, access, utilization) of food nutrition and security [8].

In the fall of 2020, the second pandemic wave reached the US, Europe, and other countries worldwide, causing additional lockdowns. Given the present uncertainties in availability, distribution, and acceptance of COVID-19 vaccines, the pandemic might continue well into 2021, and even a third wave cannot be excluded. Such repeated pandemic waves thus bring additional risk to food security. Therefore, the objective of this comprehensive review article is to evaluate the impact of the pandemic on food security. In that context, the article discusses disruptions and future threats to food security in the era of the COVID-19 pandemic and then explores the transformation of the food sector that will be necessary in order to achieve food resilience in the years to come.

## 2. Food Security during Epidemics and the COVID-19 Pandemic

Epidemics such as HIV/AIDS, Ebola, and Middle East Respiratory Syndrome (MERS) have negatively impacted food security. For example, the Ebola epidemic had a significant effect on the economies of some African countries’ agricultural production, marketing, and trade. Vulnerable populations, including children, women, the elderly, and those living in poverty, were most affected [9]. During these crises, farmers could not transport their fresh produce to local and urban markets, and much-needed aid could not be delivered to schools. The distribution chain was also impacted as supply chains were delayed, and the workforce refused to travel to infected countries due to the fear of being infected. As a result, the price of staple foods in Guinea, Liberia, and Sierra Leone increased significantly. For example, the cost of rice and cassava increased by more than 30 and 150%, respectively [10].

During the COVID-19 pandemic, a number of measures were implemented to prevent the spread of the virus and protect public health. As a consequence of lockdowns during the pandemic, households with high dependence on labor income experience a big income shock that would jeopardize the food security of these households [11]. Unfortunately, the current pandemic has precipitated an economic crisis as well as an ongoing food security and nutrition crisis, and it is still not easy to predict how COVID-19 will affect long term food security. However, previous pandemics and global crises have shown that impacts on food security can be rapid and of dramatic proportions [12]. Currently, risks, fragilities, and inequities in global food systems are arising almost daily.

The COVID-19 pandemic has been a wake-up call for food systems, which have already been sitting on a knife-edge for decades [4]. Food systems [13] incorporate all of the various food production stages including preparation, processing, distribution, consumption, and disposal. Moreover, the adequate delivery of food to consumers involves land use, agricultural inputs, infrastructure, shipping, and different actors (e.g., farmers and retailers) [3]. Thus, lockdowns and disruptions triggered by COVID-19 have complicated the interactions among these various food system elements. The whole food system, from the primary supply to the final demand, was disturbed during the COVID-19 pandemic [7]. As reported by the European Commission, the food system itself should be transformed into a more inclusive, diverse, resilient, competitive, responsible, and sustainable form [13]. The current pandemic has already affected the entire food system, presenting an extraordinary challenge with profound social and economic consequences, including compromising food security and nutrition, as outlined in the Joint Statement on COVID-19 Impacts on Food Security and Nutrition [14].

## 3. Panic Buying, Food Shortages, and Price Spikes

Table 1 presents the impacts of the pandemic on food systems. The instability caused by a shock and the related behavioral modifications can result in occasional price spikes, market and supply disruptions, and food shortages [12]. The COVID-19 pandemic affected the shopping and cooking behavior of consumers who were spending more time at home and started to cook more than ever. In addition, the uncertain consequences of the lockdowns worried consumers with regards to adequate supplies and the distribution of food products. This resulted in panic buying as many people stockpiled large amounts of products. Panic buying behavior typically originates as a result of customers purchasing more than usual not as a result of restricted food availability. Indeed, and ironically, the panic buying trigger seemed to be the moment when people were told *not* to panic. This trend was partly boosted by the media, who frequently showed pictures of empty shelves and consumers who were imitating other people’s panic-driven yet irrational and irresponsible behaviors. 

Subsequently, a surge in demand for organic and staple foods was observed similarly to what had occurred with other crises. These events included the Bovine Spongiform Encephalopathy (BSE) outbreak (2000), Severe Acute Respiratory Syndrome (SARS, 2004), and the melamine scandal (2008) that bolstered demand for organic baby food in China [15]. Indeed, food shortages and rising prices occurred in different countries (e.g., Ghana, Italy, Malaysia, and New Zealand) due to the high demand [10,16,17,18,19]. In Italy, France, Spain, Germany, Denmark, the United Kingdom, and the United States, consumers stocked up on dry yeast, which became a hard-to-find commodity [20]. In Russia, panic buying was observed the week before the self-isolation announcement, with people stocking up on buckwheat, garlic, and non-perishable foods, which were among the top-selling categories during the coronavirus panic shopping [21,22]. Subsequently, prices for staple food (e.g., sugar, tomatoes, garlic, lemon, buckwheat, and bread) prices increased by 16, 15, 9, 8, 6, and 7%, respectively) [23]. The government had advised consumers to use food delivery services, but most of them collapsed logistically as placing orders became increasingly impossible since there were no free time slots [10]. In Malaysia, the prices of cabbage and cucumbers increased by 62.5 and 300%, respectively [24]. Another consequence of the food shortage by COVID-19 panic buying could be the spread of unsafe practices, such as methanol in alcoholic beverages [25].

Food shortages and price spikes could also be related to the difficulties observed in supply chains due to border closings and quarantine measures, as well as fewer workers available for harvesting, production, logistics, and decreased production. Over the long term, labor shortages will affect the production and processing of food, particularly labor-intensive crops. For example, high-value commodities such as fruits, vegetables, and fisheries require a large amount of labor for their products, and thus, have been greatly impacted by the current situation [26]. In Germany, Britain, and Italy, rising prices were expected for certain vegetables such as asparagus and strawberries since these products are all hand-harvested by experienced workers from Eastern Europe that cannot reach the field to work [27].

According to FAO, the COVID-19 pandemic has also disrupted the food supply chain due to trade and logistics issues [12]. These disruptions reflect interruptions in the production or distribution of the products [28]. For example, due to the fact that the production of staple commodities such as wheat, maize, corn, soybeans, and oilseeds is capital intensive, the labor shortage issue will have a greater negative impact on the distribution logistics of these products and less impact on their production [26]. In Thailand, at the beginning of the COVID-19 outbreak, supermarkets were able to stay well-stocked up despite the observed panic buying. Still, a few days later, many items (especially fruit and vegetables) were missing from store shelves [10]. Commodity prices have edged up by 17.34% of the average export price (from US 481.50/tn to 565/tn) due to the higher global demand [24]. However, in this case, the main obstacle for Thai food exports was logistics, as most countries had taken lockdown measures in the aviation sector [29].

Moreover, although there is no indication that Thailand will restrict its own exports, the authorities there should consider the possibility of other countries restricting their exports. For example, the Vietnamese government announced banning new rice-export contracts at the end of March [24]. With such new export policies in place, governments of other countries may realize that they are now too dependent on foreign food supplies, and thus, should consider globalization impacts on their own food systems. Whether or not this tendency prevails will depend on the economic situation and social aspects following the post-lockdown period and the disequilibrium precipitated by the pandemic [30].

Food shortages and price increases caused by an excessive demand for particular food products have affected food availability and are disturbing for consumers. Moreover, these conditions could potentially worsen if the COVID-19 pandemic lasts for a long time. The FAO declared that panic buying and consumer stockpiling of foods reduced the donations made to food banks from supermarkets. Thus, it is essential for consumers to avoid panic buying and stockpiling in order to minimize the resultant food bank stress to food-insecure populations [31]. There is also a need to continually remind consumers that adequate food supplies are available and that the stockpiling of food is not only unnecessary but unwittingly contributes to food insecurity for many vulnerable individuals. The OECD reported that for the current pandemic situation, there is no basis for the development of a global food crisis since staple crop supplies and cereal stocks are sufficiently large. Moreover, compared to other sectors, the food sector has been less affected by business closures and movement restrictions during the pandemic. However, the pandemic poses a severe threat to food security in the poorest countries where agricultural production systems are more labor-intensive [32].

## 4. Other Impacts of the COVID-19 Pandemic

### 4.1. Impacts of the Pandemic on Agriculture

The full effect of the COVID-19 pandemic on the food chain includes not only empty shelves due to panic-buying, but also other aspects that are hard to predict in either scale or nature and yet to be seen. These impacts concern both small and commercial farming, especially in developing countries where lockdowns have led to slower food distribution systems due to border delays and the reduced ability of workers to migrate for agricultural labor and food harvesting. Unfortunately, the pre-existing food crises will continue to worsen and negatively impact the impoverished and vulnerable populations. According to the FAO, critical negative impacts on producers, transporters, processors, and consumers have been observed and will continue [12].

The problems are more intense in developing countries where many smaller farmers must transport produce and inputs by bus [33]. In particular, as the COVID-19 pandemic sweeps through the developing countries, more than 30 of them are facing a widespread famine of historical proportions, whereas, in 10 of those countries, more than a million people are on the verge of starvation [34].

The COVID-19 pandemic caused the food and agricultural sector to experience a negative downturn with an immense labor loss [35]. Labor loss prevented agricultural activities and affected supply chains. On the other hand, it caused the loss of income of the people with agricultural economies and millions of households are faced with poverty. Unfortunately, many farmers and farm laborers suicides were reported as a loss of income during the pandemic in India [36].

### 4.2. Impacts of the Pandemic on Food Supply Chains

Other impacts of the pandemic on the food chain include the following: Reduced incomes, reduced access to essential services, (e.g., veterinarians, seeds, and fertilizers) and buyers, modifications in food distribution and increased delivery needs due to closed restaurants, children losing free school meals, absenteeism due to illness across the food chain industries, increased food waste from farm to fork, as well as potential spikes in food prices due to the increased demand and slower food supply chains [19,37,38,39]. Fresh produce can accumulate without being sold which leads to food losses, loss of income, and higher food prices. Similarly, the shelf life of fresh food for the foodservice sector is very limited which leads to additional food waste [10]. Auditing, inspections, and monitoring regulations could be temporarily reduced or modified in order to expedite the movement of products. For example, in the United States, the Food and Drug Administration has issued interim guidelines that provide flexibility for various parameters such as product labeling in order to help support the food supply chain and meet consumer demand during the crisis [40]. Such administrative and regulatory changes could be supportive for some food businesses attempting to cope with lower margins and fractured supply lines, thereby addressing food quality, safety, and authenticity concerns.

These impacts highlight the need to proactively ensure contingency planning and the implementation of effective mitigation strategies and control measures, which help ensure that the health or economic crisis will not turn into a food crisis. Therefore, the recent COVID–19 health crisis could become a food crisis if adequate contingency plans are not implemented [31,41]. Indeed, an integral approach from governmental and research bodies, as well as the industry and consumers is essential in order to provide a safety net for the most vulnerable populations and to ensure that the food supply chain operates efficiently. This approach includes health and safety measures [42] and social distancing [43], as well as government interventions, investments, and reduced tax policies in the agricultural sector [26]. Other relevant measures include purchasing agricultural products from small farmers and shorter supply chains [44], development of e-commerce platforms, and mobilization of non-governmental food banks whose staff have the technical knowledge and experience to deliver food efficiently [26]. However, those actions will not be sufficient unless implemented in a timely and coordinated manner. For instance, local food crop production can only fulfill less than one-third of the world’s population [45]. Despite the pandemic, the food supply chain must keep working, and, at the same time, adequate measures must be in place to ensure the highest standards in order to prevent further spreading of the virus. Unfortunately, the supply chain is sometimes weak, and many products have been lost since the demand is not adequate enough to purchase the products at their regular price [26].

Moreover, the food chain is complex and involves many factors from farm-to-table. This complexity can create gaps among the producer, consumer, and the product itself. Consumers’ food choices are influenced by the following factors: Price, nutrition, health benefits, quality, origin, seasonality, emotions, habit, labeling, access, sensory characteristics, culture, personal preference, environmental footprint, and previous positive experience and information. Other factors include a preference for organic products, choosing local products, animal welfare, sourcing ingredients for planned meals, advertisements, minimal processing, and shelf-life [46,47,48,49].

### 4.3. Impacts of the Pandemic on Packaging

The COVID-19 pandemic has also affected the packaging industry in different sides such as increasing the consumer awareness on the hygiene and safety of packaging materials, increasing the digital printing, packaging for e-commerce shipments, as well as rethinking the materials and design requirements of sustainable packaging [50,51]. For achieving sustainable goals, many packaging companies had developed reusable innovative packaging technologies. However, the pandemic caused by a coronavirus affected consumer behaviors due to the concerns on hygiene. In addition, the safety of reusable packaging temporarily halted the packaging industry’s improvements on a sustainable supply chain [50]. For example, Starbucks temporarily suspended the use of personal cups rather than single use paper cups at its stores around the world in response to the COVID-19 pandemic [52,53], since concerns on hygiene have a greater priority than environmental concerns. In that context, packaging companies should transform packaging design taking into consideration the main requirements including sustainability, heightened hygiene and safety concerns of the consumers as well as, design for e-commerce, ship-ready design, and direct-to-consumer models [51].

## 5. Food Loss and Waste

The COVID-19 pandemic may also affect the lost and wasted food on a short and long term basis [32]. Consumer waste has arisen mainly from the over-buying trend and improper storage of high quantities of foods. On the other hand, food supply chains were disrupted due to road closures which caused an accumulation of products, resulting in the increased levels of food loss and waste [26]. In order to reduce food waste, the EU Platform on Food Losses and Food Waste shared the food loss and waste prevention actions taken by EU Member States of the EU in the context of this unprecedented crisis [54]. Likewise, many governments warned citizens that no widespread food shortages had been observed and informed them regarding how to plan shopping and food storage in order to modify their consumption habits [32,54]. The mobilization of private charities and community-based groups to distribute food during the lockdown could solve several problems concomitantly by helping to reduce food waste while supporting people in need [3]. A similar practice was implemented by several cooperatives and municipalities that collected surplus food from school cafeterias and restaurants and redistributed it to the low-income and other vulnerable groups [54]. Such alternative supply channels for handling potential surpluses or potential food loss and waste that have resulted from the closure of restaurants, schools, hotels, and catering businesses have been significant and appreciated resources during the pandemic [32].

In general, modern food supply chains have focused on reducing food loss and waste (basically to minimize cost), and subsequently, environmental impacts. However, the unpredicted spike in food demand as a result of COVID-19 control measures has led to empty shelves. This massive shock to well-organized food supply chains highlighted the need for increased consumer education. Many modern technologies proposed helping to monitor food production and consumption (aiming at reducing food loss and waste), which can be used to ensure a reliable, uninterrupted food supply during these challenging times.

## 6. Food Resilience

Any organized system aims to reach an optimal operational state and remain stable. However, this approach is ideal and often not possible in our fast-changing world where systems stability depends on the outbreak frequency of extreme events rather than typical conditions. The greater the attempt to optimize the elements of a complex system, the more diminished the resilience. An external change during the optimal state could result in disturbances and, subsequently, a more vulnerable system [55]. The current food systems could be disrupted due to many factors, including urbanization, population aging, and occasional shocks such as economic crises, natural disasters due to climate change, and unpredicted responses to extreme events [56]. Therefore, food systems should be more resilient in order to adapt to extreme situations such as the one we are living in today [13], and system weaknesses, choke points, vulnerabilities, and critical services should be well-refined [32].

Resilient food systems could contribute to food security and, ultimately, to sustainable food systems [57], as those are complementary concepts [58]. In particular, sustainability concerns the capacity to achieve today’s goals without compromising the future ability to achieve them, and resilience is the dynamic capacity to continue achieving goals despite shocks and disturbances [59,60]. Thus, the food systems could be sustainable when their elements are flexible enough to absorb shocks and mitigate damages as a result of changes in their natural conditions [58,61,62].

In complex systems, sudden shifts could surprise us, but working at the crossroads of these emerging fields offers new approaches to anticipate critical transitions [63]. In the case of the COVID-19 pandemic, food security is affected [6], showing that our food systems are not resilient enough to adapt to severe changes such as economic crises [3] and climate change [64]. Although different, the pandemic and climate risk share common characteristics as both of them represent physical shocks, systemic, non-stationary, and regressive changes. Therefore, the current pandemic provides us with a preview of future challenges to supply and demand, disruption of food supply chains, and amplification mechanisms due to climate change. Moreover, the measures taken for each could result in an enhanced understanding of the other one. For instance, climate-resilient infrastructures could increase economic and environmental resiliency [65].

This pandemic and the occurring disruptions offer a unique opportunity to learn more about the fragility and critical points of the system in order to increase preparedness for future disruptions [66]. Likewise, it has created opportunities for innovations [67], e.g., the need for social distancing, remote work, and improved delivery systems leading to the development of mobile applications and internet and communication technologies that can also be implemented with regard to food loss and waste [3]. The conversion of farms to carbon and organic farming could contribute to a more resilient urban food system [68]. However, this will not completely solve food insecurity and diet-related problems. Likewise, there is a need for increased policy intervention with regard to dietary patterns, e.g., more regulation of the ingredients in junk food and actions to make fresh food more accessible and affordable [69]. Within the global food syndemic, there are opportunities to develop healthy eating patterns for consumers’ wellness based on products that address food insecurity, malnutrition, and obesity.

Huff et al. [70] predicted the pandemic’s effect on the US-food system, showing that a severe event resulting in a higher than 25% reduction in labor availability could lead to significant food shortages [70]. Therefore, it is essential to limit the disruption of critical infrastructures during a pandemic or a climate crisis in order to maintain an adequate movement of food and water supplies which are critical for the survival and health of society. Progress can be achieved by accelerating investments in data systems in order to enhance consumer confidence in supplies during disruptions [32]. The preparation of food systems against potential hazards is also essential [4]. Mitigation measures such as enhanced biosecurity arrangements to manage sanitary and phytosanitary risks should be considered [32]. In addition, system changes should result in a shift from an optimized shorter-term performance model to an approach that ensures equally longer-term resiliency [65,71].

The COVID-19 pandemic has showed the importance of resilient agri-food system. The agricultural and food systems cannot be resilient if they are not sustainable. Therefore, it is very important to transform food systems using new technologies and scientific discoveries, combined with an increasing public awareness and demand for sustainable food [72].

## 7. Transformation of the Food Sector 

Food security depends not only on food availability but also on food access and utilization. Subsequently, significant improvements in the global food system and forest/land governance are required [69]. The 47th Session of the UN Committee on World Food Security recommended joint action towards a comprehensive transformation of global agri-food systems, to make them more inclusive, resilient, and sustainable [73]. The cornerstones of the transformation are innovation [74] and productivity [75], together with the way in which the biomass for food and feed is produced, processed, and consumed [3]. During the transformation, it is essential to adopt an integrated approach that includes food waste reduction and valorization [3,76] and a shift to the climate-neutral economy [77]. This approach would provide new perspectives for farmers and rural areas, reducing greenhouse gas (GHG) emissions, as well as improving carbon, nitrogen, and phosphorous circularity and overall land-use efficiency [69].

Among the urgent challenges for the food industry in the post-COVID era is the development of competitive, sustainable, and affordable products that promote and enhance health. Researchers are not only seeking food bioactive compounds [78], but also recovering these compounds from food processing by-products in order to replace synthetic additives with natural ingredients that possess health benefits [79,80,81,82,83,84]. Additional energy-efficient and sustainable processing technologies are needed to support these efforts [85,86,87,88,89,90,91,92]. Phenotyping and gene editing have also resulted in new opportunities. Advances in precision fermentation, synthetic biology, and microbiology will soon result in food produced in laboratories, e.g., lab-grown meat and novel alternative protein sources [69]. Consumers, governments, and companies will also play a vital role in the transformation by helping in changing dietary behaviors to include healthier choices such as plant-based foods and less meat. The latest would eliminate food overconsumption, end malnutrition, and finally improve health [93,94]. Moreover, there is a need to develop bioanalytical tools to ensure food and environmental safety during this transformation [95].

The transformative food sector requires different policies that reconsider the elements of our food systems and facilitate the relations between them. Taking the EU as an example, the Biodiversity Strategy [96] and the EU Farm to Fork [97] strategies have highlighted the transformation of the food system by reducing the use of fertilizers and pesticides and promoting carbon neutrality, as well as the increase in organic farming and protected agricultural areas. In addition, many shifts must take place simultaneously at the societal level. For instance, spending more on local food should become a priority to shrink the urban-rural gap considering potential energy savings from the transportation expenditure [69]. Moreover, consumer confidence in the safety of the agro-food system should be taken into consideration by enhancing government communication strategies [32]. 

The agricultural self-sufficiency of people, cities, and countries should also increase, whereas agriculture and aquaculture should be resilient against market failure and climate change. In such a system, healthy societies will grow, and this system could be achieved by human-centered and nature-based design [67]. Emergency cash flow and economic measures for the food supply chain are necessary in order to support the needs of farmers, fishers, and agri-food businesses [98]. For example, governments should consider crowdfunding for local bioeconomic investments as part of their regional development funds and recovery plans. Finally, the implementation of technology disruptions is necessary in order to transform the food sector in the new era. Industry 4.0 applications, blockchain in the food supply chain, and Internet and Communication Technologies are the innovations with the highest potential in the new era. There is also an equally pressing need to exploit social marketing to understand consumers’ attitudes in order to adapt to new norms forged by the COVID-19 pandemic, where there is a significant gap in knowledge for decision making [99].

## 8. Conclusions

The COVID-19 pandemic ushered in a new era in the food supply chain as we are still trying to figure out the consequences on humanity, the economy, food safety, and food security [3]. From panic buying, food shortages, and price spikes, to other social and economic impacts, as well as food loss and waste issues, this crisis has shown that our food systems are fragile and need to be redesigned in order to increase food security. Improving food systems to make them more sustainable and resilient should be more than ever an urgent priority. Over the next decades, both the global population and urbanization will grow, pandemics will occur more often, and climate change will intensify. As a result, our societies’ transitions towards sustainable development and a climate-neutral economy must be based on resilient food systems. Such systems should include contingency plans and mitigation strategies based on innovations, productivity issues, and consumption patterns that would allow rapid response and adaptation to extreme events, as well as ensuring that inevitable crises will minimally affect the food chain and our most vulnerable populations.

## Figures and Tables

**Table 1:** Impact of the COVID-19 pandemic on the food systems.

Sector	Impact
Production	Decreased availability of food Price spikes Shortage of inputs and labor Demand collapsed due to lockdowns Disposal of perishable foods and increased food waste amounts
Processing	Price spikes Innovations gap due to lack of investments Demand collapsed due to lockdowns Income reduction and unemployment of workers Disposal of perishable foods and increased food waste amounts
Retailing	Food shortage due to panic buying The rapid development of e-commerce and direct connection of farmers with consumers Reduced local availability Disruption of transportation flows and wholesale markets
Consumption	Demand collapsed due to lockdowns The rapid development of home delivery Food insecurity for vulnerable individuals Income reduction and unemployment of workers in the catering sector Change in eating behaviors
